# Genetic Basis of Early Onset Atrial Fibrillation in Patients without Risk Factors

**DOI:** 10.3390/jcdd10030104

**Published:** 2023-02-28

**Authors:** Irina Rudaka, Baiba Vilne, Jekaterina Isakova, Oskars Kalejs, Linda Gailite, Dmitrijs Rots

**Affiliations:** 1 Scientific Laboratory of Molecular Genetics, Rīga Stradiņš University, LV-1007 Riga, Latvia; 2 Latvian Cardiology Centre, Pauls Stradiņš Clinical University Hospital, LV-1002 Riga, Latvia; 3 Bioinformatics Laboratory, Rīga Stradiņš University, LV-1007 Riga, Latvia

**Keywords:** early-onset atrial fibrillation, genetics, next-generation sequencing, pathogenic variants

## Abstract

Background: Atrial fibrillation (AF) is the most common arrhythmia and typically occurs in elderly patients with other cardiovascular and extracardiac diseases. However, up to 15% of AF develops without any related risk factors. Recently, the role of genetic factors has been highlighted in this particular form of AF. Aims: The aims of this study were to determine the prevalence of pathogenic variants in early-onset AF in patients without known disease-related risk factors and to identify any structural cardiac abnormalities in these patients. Materials and Methods: We conducted exome sequencing and interpretation in 54 risk factor-free early-onset AF patients and further validated our findings in a similar AF patient cohort from the UK Biobank. Results: Pathogenic/likely pathogenic variants were found in 13/54 (24%) patients. The variants were identified in cardiomyopathy-related and not arrhythmia-related genes. The majority of the identified variants were TTN gene truncating variants (TTNtvs) (9/13 (69%) patients). We also observed two TTNtvs founder variants in the analysed population—c.13696C>T p.(Gln4566Ter) and c.82240C>T p.(Arg27414Ter). Pathogenic/likely pathogenic variants were found in 9/107 (8%) individuals from an independent similar AF patient cohort from the UK Biobank. In correspondence with our Latvian patients, only variants in cardiomyopathy-associated genes were identified. In five (38%) of the thirteen Latvian patients with pathogenic/likely pathogenic variants, dilation of one or both ventricles was identified on a follow-up cardiac magnetic resonance scan. Conclusions: We observed a high prevalence of pathogenic/likely pathogenic variants in cardiomyopathy-associated genes in patients with risk factor-free early-onset AF. Moreover, our follow-up imaging data indicate that these types of patients are at risk of developing ventricular dilation. Furthermore, we identified two TTNtvs founder variants in our Latvian study population.

## 1. Introduction

Atrial fibrillation (AF), the most common cardiac arrhythmia, is increasing in incidence and prevalence. This trend is associated with enhanced AF detection, an augmentation in the occurrence of AF-related risk factors and improved treatment of cardiovascular diseases [1]. Most commonly, AF is secondary or a complication of other diseases and/or structural changes in the heart [2,3]. While the majority of AF cases are attributable to well-established disease risk factors (e.g., coronary heart disease, heart failure, hypertension, diabetes, hyperthyroidism, chronic kidney disease, etc.), up to 15% of patients develop AF in their absence [4]. This form of the disease used to be referred to as ‘lone AF’. However, due to the lack of united criteria for this form and also uncertainty regarding which investigations should be performed to exclude all possible causes for AF, it is currently recommended to avoid the usage of this term [5,6]. Hence, in the present study, we have chosen to use the term ‘early-onset AF without risk factors’ (EOAF).

Recently, genetic factors have been highlighted to play a significant role in the aetiology of AF, with both common and rare variants being implicated [7]. Rare pathogenic variants in genes encoding ion channel subunits and structural proteins have been found to be causative. However, the diagnostic yield is low and highly variable across studies, ranging from 1% to 24% [8,9,10,11,12]. Pathogenic variants in the same genes have previously been described in association with cardiomyopathies and primary electrical disorders of the heart [12,13]. Additionally, it has been suggested that early-onset AF patients without any significant comorbidities and a normal initial clinical investigation, who carry rare variants in cardiomyopathy-related genes, have an increased risk of developing structural abnormalities of the heart that are detected only using cardiac magnetic resonance (CMR) imaging and/or computed tomography [11]. However, the amount of currently available data is sparse and at present, it is not known whether AF caused by pathogenic variants in hereditary cardiac disorder-related genes is a separate entity, an early manifestation of structural heart disease or an expression of atrial cardiomyopathy. Furthermore, there are currently no recommendations for the selection of AF patients suitable for genetic testing and EOAF case follow-up/clinical care. Therefore, in this study, we aimed to determine the prevalence of causative pathogenic variants in EOAF cases using exome sequencing and to identify any structural cardiac abnormalities in these patients.

## 2. Materials and Methods

### 2.1. Study Population

Our study population comprised 54 symptomatic EOAF index patients without any AF-related risk factors. They were recruited at tertiary care clinic specialising in cardiac disorders in Latvia. Early-onset AF was defined as AF occurring before the age of 65. Familial AF was defined as documented AF in at least one first- or second-degree relative reported by the index case. All patients underwent an initial transthoracic echocardiography evaluation according to the standard protocol adopted by the Latvian Society of Cardiology, as well as laboratory testing to exclude the presence of risk factors (i.e., creatinine, haemoglobin A1c, thyroid-stimulating hormone, free thyroxine). Coronary heart disease had previously been excluded in all patients using imaging modalities (computer tomography angiography or invasive angiography). Furthermore, patients were excluded if they had any of the following comorbidities associated with AF: valvular heart disease, hypertension, cardiomyopathy, heart failure, diabetes, thyroid dysfunction (hyper- or hypothyroidism), chronic kidney disease, chronic obstructive pulmonary disease, systemic disease, malignancy, alcohol abuse, sedentary lifestyle or regular vigorous exercise. Each patient’s AF-related risk factor profile was assessed with a clinical investigation and from previous medical records.

For cases with an identified pathogenic/likely pathogenic variant (according to the ACMG guidelines [14]), follow-up CMR imaging was performed. The images were analysed according to the standard protocol adopted by the Latvian tertiary care clinic specialising in cardiac disorders.

Additionally, to confirm the findings from our Latvian study population, an independent similar AF patient cohort from the UK Biobank [15,16] was analysed (107 EOAF cases; see filtering strategy below). The criteria used for the definition and inclusion of AF cases were similar to those used for our study cohort and are detailed in Supplementary Table S1.

Moreover, an in-house database of exome sequencing data derived from patients with suspected hereditary cardiac (e.g., cardiomyopathies) and non-cardiac (e.g., neuropathies, etc.) phenotypes—who had been referred to our laboratory for genetic evaluation—was available for comparison purposes for any EOAF patient findings.

The study protocol was approved by the Latvian Central Medical Ethics Committee (Permission No.: 1/16-05-09 approved 09.05.2016) and was performed according to the Declaration of Helsinki. All AF patients gave written informed consent for participation in the study.

### 2.2. Genetic Analysis

All AF patients were profiled using exome sequencing: 6 patients using TruSight One capture kit (clinical exome, *n* = 4800 genes; Illumina, San Diego, CA, USA), 15 patients using Twist Human Core Exome Kit (Twist Bioscience, San Francisco, CA, USA) sequenced on NextSeq 500 platform (Illumina) with 75PE reads, and 33 patients using Twist Comprehensive Exome Panel (Twist Bioscience) sequenced on NovaSeq 6000 platform (Illumina) with 100PE reads (service provided by CeGaT, Tübingen, Germany).

After sequencing, raw reads were mapped to the GRCh38 reference with no alternate contigs using BWA-MEM v0.17 [17] with subsequent duplicate removal using sambamba [18]. Short variant calling for nuclear DNA was performed using DeepVariant v0.7.2 [19] or a newer version, and mtDNA variants were called using Strelka2 v2.9.0 [20] with continuous ploidy to account for possible heteroplasmy. Further, variants were annotated and analysed using the BaseSpace Variant Interpreter platform (Illumina), analysing variants in AD, AR, XL and mitochondrial mode of inheritance, using a virtual gene panel containing 349 genes previously associated with inherited cardiac disorders (including arrhythmia and cardiomyopathies; Supplementary Table S2). Copy number variants were called using XHMM and ExomeDepth tools [21] as implemented in the Ximmer tool and annotated using AnnotSV [22].

Variant interpretation for all individuals (including the in-house and UK Biobank cohorts) was performed according to the ACMG variant interpretation guidelines [14] and also using cardiodb [23] for the TTN gene. This analysis was conducted by at least two people with experience in cardiac conditions, with one of them being a certified molecular geneticist.

To ensure that no familial cases were analysed, the relatedness of the samples was excluded with VCFtools using the relatedness2 option [24]. For the haplotype analysis, genomic variant calling files (gVCFs) were combined into a multisample VCF containing all AF and additional cardiomyopathy patients (*n* = 115). The VCF was filtered to remove indel variants, as well as variants with missing genotypes in more than half of the individuals. Further, the VCF was manually inspected in IGV [25].

For validation of the findings from our Latvian study population, we used data from the UK Biobank [16], a large population-based prospective cohort study from the United Kingdom with deep genetic and phenotypic data on approximately 500,000 individuals aged 40 to 69. We downloaded the joint-call exome data (Field 23156) for 200,000 participants in pVCF format (accessed on November 2020) and used an EOAF case definition similar to the one used for the Latvian cohort (Supplementary Table S1). We further performed individual-level filtering by removing missingness or heterozygosity outliers, participants with self-reported vs. genetically inferred sex mismatches or putative sex chromosome aneuploidy, individuals that were not of European ancestry and individuals having withdrawn their consent at the time of analysis. We also identified closely related participants (kinship coefficient > 0.088, i.e., first- or second-degree relative pairs), preferentially retaining EOAF cases. After the filtering, 107 individuals with exome sequencing data were available for analysis.

## 3. Results

### 3.1. Patient Analysis of the Latvian Study Population

In total, this study included 54 EOAF patients, of which 44 (81%) were male. The mean age of the AF patients at recruitment was 46.8 ± 10.7 years, and the mean age at AF onset was 42.7 ± 12.0 years. There was a positive family history (presence of AF) in 21 (39%) of the 54 cases. No data on cardiomyopathy, heart failure, sudden cardiac death or stroke in the family were available. An overview of the study participants is shown in Table 1. 

Pathogenic (P) and likely pathogenic (LP) variants were found in 13 (24%) of the 54 patients (Table 1), indicating that they very likely have monogenic AF. Sex, positive AF family history and age at AF onset did not differ significantly between these putative monogenic EOAF patients and the remaining EOAF patients without identified P/LP variants (*p* = 0.05, 0.2 and 0.27, respectively).

Interestingly, all observed variants were located in cardiac structural and developmental genes, which primarily play a role in cardiomyopathy and congenital heart disease development [26,27,28,29,30]. No rare P or LP variants previously described in arrhythmia-associated genes were observed in our cohort. Nine (17%) of the 54 patients had LP truncating variants in the TTN gene (TTNtvs), making it the most prevalent causative gene in this cohort.

Five patients were positive for the TTN variant NM_001267550.2:c.13696C>T p.(Gln4566Ter). This variant was also present in two independent and unrelated patients in our in-house database; one with arrhythmogenic cardiomyopathy (ACM) and one with dilated cardiomyopathy (DCM). Furthermore, the TTN variant NM_001267550.2:c.82240C>T p.(Arg27414Ter) detected in one of our EOAF patients was also present in two independent and unrelated patients with DCM in our in-house database. These two variants were not identified in the exome sequencing data of the approximately 300 individuals with no cardiac disorders in our in-house database, thus excluding the possibility that they are common population-specific variants. All the study subjects, as well as the aforementioned ACM and DCM patients, with recurrent TTNtvs were unrelated (relatedness phi score < 0.04). Moreover, for both of the variants (p.(Gln4566Ter) and p.(Arg27414Ter)), there appears to be a distinct haplotype shared among the individuals with the same variant. Although haplotype phasing from exome sequencing data for heterozygous variants is not optimal, we suggest that c.13696C>T p.(Gln4566Ter) and c.82240C>T p.(Arg27414Ter) are founder variants in the Latvian population that cause not only AF but also ACM and DCM.

### 3.2. UK Biobank Data Analysis

To validate the findings from our Latvian study population, an independent similar AF patient cohort from the UK Biobank was analysed. After individual-level quality control and filtering, we were able to extract 107 EOAF cases. The interpretation of variants was conducted in a similar way to our clinical cases. As a result, a monogenic cause (i.e., carrying a P/LP variant in one of the 349 genes associated with hereditary cardiac disorders) was identified for 9 (8%) of the 107 individuals. No recurrent variants were observed among these individuals. In correspondence with our Latvian patients, only variants in cardiac structural or developmental genes were identified (Table 2).

### 3.3. Imaging in Monogenic AF Patients

CMR imaging was performed on study patients with monogenic AF (P/LP variant carriers; Table 1) at a follow-up visit. In 5 (38%) of the 13 P/LP-carrying patients, dilation of the left or both ventricles was observed despite having a normal cardiac ultrasound at recruitment. One of these patients carried an LMNA variant—c.976T>A p.(Ser326Thr). Notably, the four others all carried the same founder variant—TTN c.13696C>T p.(Gln4566Ter). The remaining patient with this founder variant displayed normal CMR imaging results. In addition, the patient with a BAG3 variant (c.870dupC p.(Ser291LeufsTer28)) exhibited increased T1 mapping intensity of septal wall segments.

## 4. Discussion

In this study, we performed diagnostic-level exome sequencing in 54 EOAF cases from the Latvian population. We observed a high prevalence of pathogenic/likely pathogenic variants exclusively in cardiac structural or developmental genes. We did not detect any variants in arrhythmia-related genes. These findings from our Latvian study population were replicated in an independent similar EOAF patient cohort from the UK Biobank (16). Moreover, a high prevalence of TTN truncating variants across individuals with confirmed aetiology was observed in our study population. Furthermore, we identified two TTNtvs founder variants in the Latvian study population that may be responsible for the development of not only AF but also ACM and DCM.

While we have focused on large-effect size rare pathogenic variants, it is also known that common variants (e.g., identified using GWAS) could contribute to the risk of developing EOAF [7]. Several recent studies have reported a high prevalence of rare causative variants in early-onset (before the age of 66 years) AF patients [7,8,9,10,11,12]. Interestingly, in the majority of cases, the disease-causing variants were found in cardiomyopathy-related genes; only a few were located in arrhythmia-related genes [11,31]. However, the diagnostic yield across these studies is highly variable (1% to 24%) due to different inclusion criteria and the diagnostic methods being employed [8,9,10,11,12]. Of note, the EOAF diagnostic yield in our Latvian study population was 24%, whereas in the UK Biobank cohort, it was 10%. The difference between these two diagnostic yields may be due to, at least in part, the presence of two TTNtvs founder variants and the high prevalence of cases with a positive family history in our study population.

TTNtvs are known to be associated with the aetiopathogenesis of cardiomyopathies, primarily with dilated cardiomyopathy [32,33,34]. There is also strong evidence of TTNtv causality in the development of AF. For instance, an analysis of 2781 early-onset (before the age of 66 years) AF cases found a statistically significant association between TTNtvs and EOAF [9]. Furthermore, an exome-wide gene-based burden analysis of AF cases and controls from the UK Biobank database found that only rare TTNtvs reached statistical significance across all AF cases regardless of the age at onset and risk factors [7]. In our AF cohort, TTNtvs accounted for 17% of cases. However, the majority of previous studies have reported a lower prevalence (2–10%) of TTNtvs among AF patients [31,35,36], although a TTNtv prevalence of 16% has been reported in a cohort of very early-onset (<45 years) AF patients with structurally and functionally normal hearts [11]. We believe that founder variants play a role in the common occurrence of TTNtvs among our EOAF patients. Interestingly, one of the TTNtv founder variants, c.13696C>T p.(Gln4566Ter), has also been observed in the Estonian population (based on gnomAD), which has a close historical and geographical relationship with Latvia [37]. Therefore, this variant is probably a founder variant in the Estonian population as well. To date, the literature has information on only a single TTNtv founder variant among DCM patients in the Netherlands [38].

The presence of TTNtvs impacts the structural and functional remodelling of the heart and the natural course of AF. In patients with TTNtv-related AF, a significantly increased left atrial late gadolinium enhancement (an indicator of the presence of fibrosis) has been identified, along with a reduced left ventricular ejection fraction and chamber dilation [11,39,40]. Notably, in our cohort, dilation of one or both ventricles was observed in 4/9 TTNtv-positive individuals upon detailed CMR assessment. Left ventricular dilation was also observed in a patient with LMNA-related AF. Furthermore, a patient with a likely pathogenic variant in the BAG3 gene showed increased segmental T1 mapping suggestive of structural myocardial alteration. The identification of structural heart abnormalities raises the suspicion that EOAF may in fact be an initial electrical manifestation of cardiomyopathy. Therefore, our data suggest that in these types of patients, a detailed examination of the heart’s morphology and function (using CMR imaging), as well as regular follow-ups are required, especially in TTNtv-related AF cases. Yoneda et al. recently showed that the presence of disease-causing variants in cardiomyopathy- and arrhythmia-associated genes are related to a higher mortality risk in patients with early-onset AF, thus revealing this disease entity to be less benign than previously thought [41]. However, data on the impact of structural gene variants on AF burden, quality of life and risk of stroke is still missing. Taking these findings and our data together, genetic testing could play an important role in risk stratification in early-onset AF patients and, therefore, should be considered in clinical settings for patient diagnostics [42].

## 5. Conclusions

In conclusion, we have shown that EOAF patients have a high prevalence of cardiomyopathy-related pathogenic/likely pathogenic variants, and these patients are at risk of developing cardiomyopathy. We also identified two TTNtv founder variants in our Latvian study population that may be responsible for the development of not only AF but also ACM and DCM.

## Figures and Tables

**Table 1:** Description of the atrial fibrillation patients and their genetic testing results.

No.	Sex	Age at Examination	AaO ^†^	Family History of AF	P/LP Variant Identified in This Study	Imaging Follow-Up ^‡^	Time to Follow-Up ^∇^, Months
1.	Male	63	60	No	No	NA	NA
2.	Male	32	27	No	No	NA	NA
3.	Male	55	53	Mother	*LMNA* NM_170707.4 c.976T>A p.(Ser326Thr)	Left ventricular dilation	39
4.	Male	46	46	No	No	NA	NA
5.	Male	48	48	No	No	NA	NA
6.	Male	56	48	No	No	NA	NA
7.	Male	49	44	No	No	NA	NA
8.	Female	53	45	No	*TTN* NM_001267550.2 c.13696C>T p.(Gln4566Ter)	Biventricular dilation	23
9.	Male	62	61	No	No	NA	NA
10.	Male	43	37	No	*TTN* NM_001267550.2 c.13696C>T p.(Gln4566Ter)	Biventricular dilation	35
11.	Male	32	30	No	*TTN* NM_001267550.2 c.13696C>T p.(Gln4566Ter)	No structural or morphological abnormalities	5
12.	Male	43	43	Mother	No	NA	NA
13.	Male	28	18	Father, paternal grandmother, maternal grandmother	*TTN* NM_001267550.2: c.13696C>T p.(Gln4566Ter)	Left ventricular dilation	10
14.	Male	44	39	Brother	No	NA	NA
15.	Male	55	39	Mother and maternal grandmother	*TTN* NM_001267550.2: c.13696C>T p.(Gln4566Ter)	Biventricular and biatrial dilation	12
16.	Female	57	55	Mother	*TTN* NM_001267550.2: c.85223C>Gp.(Ser28408Ter)	No structural or morphological abnormalities	51
17.	Female	65	58	No	*TTN* NM_001267550.2: c.82240C>T p.(Arg27414Ter)	No structural or morphological abnormalities	15
18.	Female	53	52	Mother	*TTN* NM_001267550.2: c.3034C>T p.(Arg1012Ter)	No structural or morphological abnormalities	10
19.	Male	50	41	Son	*TTN* NM_001267550.2: c.95561G>A p.(Trp31854Ter)	No structural or morphological abnormalities	12
20.	Female	58	56	No	No	NA	NA
21.	Male	44	41	Mother	No	NA	NA
22.	Male	56	55	No	No	NA	NA
23.	Male	47	44	Mother	No	NA	NA
24.	Male	55	55	Father	No	NA	NA
25.	Male	48	45	No	No	NA	NA
26.	Male	65	64	No	No	NA	NA
27.	Male	65	64	No	No	NA	NA
28.	Female	64	64	No	No	NA	NA
29.	Male	61	61	Brother	No	NA	NA
30.	Male	32	30	Father	No	NA	NA
31.	Male	38	35	No	No	NA	NA
32.	Male	49	35	Mother and maternal grandmother	No	NA	NA
33.	Male	49	37	Mother	No	NA	NA
34.	Male	46	46	Mother	No	NA	NA
35.	Female	52	43	Father	No	NA	NA
36.	Male	46	45	No	No	NA	NA
37.	Male	50	48	No	No	NA	NA
38.	Male	35	31	No	No	NA	NA
39.	Female	47	41	Mother	No	NA	NA
40.	Male	40	39	Maternal mother	No	NA	NA
41.	Male	28	25	No	*RBM20* NM_001134363.3: c.1898C>T p.(Pro633Leu)	No structural or morphological abnormalities	6
42.	Male	31	21	Sister	*BAG3* NM_004281.4: c.870dupC p.(Ser291LeufsTer28)	Increased T1 mapping intensity of S8 and S9 segments of septal wall	4
43.	Female	48	43	No	*NKX2-5* NM_004387.4: c.325G>T p.(Glu109Ter)	No structural or morphological abnormalities	8
44.	Male	45	42	No	No	NA	NA
45.	Male	32	31	No	No	NA	NA
46.	Male	34	33	No	No	NA	NA
47.	Male	48	47	No	No	NA	NA
48.	Male	61	60	No	No	NA	NA
49.	Male	26	14	Father	No	NA	NA
50.	Male	24	23	No	No	NA	NA
51.	Female	49	47	No	No	NA	NA
52.	Male	37	37	No	No	NA	NA
53.	Male	39	29	No	No	NA	NA
54.	Male	50	41	No	No	NA	NA

^†^ AaO—age at AF onset. ^‡^ Cardiac magnetic resonance imaging was performed only in cases with identified pathogenic/likely pathogenic (P/LP) variants based on ACMG criteria. ^∇^ Follow-up time from initial assessment.

**Table 2:** P/LP variants and clinical data of 107 EOAF patients from the UK Biobank.

No.	Variant	Sex	Age at AF Onset
1.	*TNNT2* NM_001276345.2: c.838G>A p.(Asp280Asn)	Female	45
2.	*TTN* NM_001267550.2: c.72223_72224insG p.(Lys24075ArgfsTer12)	Male	62
3.	*GATA5* NM_001276345.2: c.781G>A p.(Glu261Lys)	Female	58
4.	*TNNI3* NM_000363.5: c.454G>A p.(Asp152Asn)	Female	62
5.	*ACTN2* NM_001103.4: c.2368-2A>G p.?	Male	58
6.	*TTN* NM_001267550.2: c.37543+1G>A p.?	Male	62
7.	*PKP2* NM_001005242.3: c.1177C>T p.(Gln393Ter)	Male	52
8.	*MYH7 * NM_000257.4: c.427C>T p.(Arg143Trp)	Female	60
9.	*MYBPC3* NM_000256.3: c.1484G>A p.(Arg495Gln)	Female	64

## Data Availability

Data is available upon request.

## Supplementary Materials

The following supporting information can be downloaded at: https://www.mdpi.com/article/10.3390/jcdd10030104/s1, Table S1: Definition of early onset risk factor-free cases in UK Biobank database; Table S2: List of genes included into analysis.

## References

[1] Schnabel R.B., Yin X., Gona P., Larson M.G., Beiser A.S., McManus D.D., Newton-Cheh C., Lubitz S.A., Magnani J.W., Ellinor P.T. (2015). 50 year trends in atrial fibrillation prevalence, incidence, risk factors, and mortality in the Framingham Heart Study: A cohort study. Lancet.

[2] Rahman F., Kwan G.F., Benjamin E.J. (2014). Global epidemiology of atrial fibrillation. Nat. Rev. Cardiol..

[3] Anumonwo J.M.B., Kalifa J. (2014). Risk Factors and Genetics of Atrial Fibrillation. Cardiol. Clin..

[4] Kloosterman M., Oldgren J., Conen D., Wong J.A., Connolly S.J., Avezum A., Yusuf S., Ezekowitz M.D., Wallentin L., Ntep-Gweth M. (2020). Characteristics and outcomes of atrial fibrillation in patients without traditional risk factors: An RE-LY AF registry analysis. EP Eur..

[5] Wyse D.G., Van Gelder I.C., Ellinor P.T., Go A.S., Kalman J.M., Narayan S.M., Nattel S., Schotten U., Rienstra M. (2014). Lone atrial fibrillation: Does it exist?. J. Am. Coll. Cardiol..

[6] Kirchhof P., Benussi S., Kotecha D., Ahlsson A., Atar D., Casadei B., Castella M., Diener H.C., Heidbuchel H., Hendriks J. (2016). 2016 ESC Guidelines for the management of atrial fibrillation developed in collaboration with EACTS. Eur. Heart J..

[7] Choi S.H., Jurgens S.J., Weng L.C., Pirruccello J.P., Roselli C., Chaffin M., Lee C.J., Hall A.W., Khera A.V., Lunetta K.L. (2020). Monogenic and Polygenic Contributions to Atrial Fibrillation Risk: Results from a National Biobank. Circ. Res..

[8] Vad O.B., Paludan-Müller C., Ahlberg G., Kalstø S.M., Ghouse J., Andreasen L., Haunsø S., Tveit A., Sajadieh A., Christophersen I.E. (2020). Loss-of-Function Variants in Cytoskeletal Genes Are Associated with Early-Onset Atrial Fibrillation. J. Clin. Med..

[9] Choi S.H., Lu-Chen W., Roselli C., Lin H., Haggerty C.M., Shoemaker M.B., Barnard J., Arking D.E., Chasman D.I., Albert C.M. (2018). Association between titin loss-of-function variants and early-onset atrial fibrillation. JAMA.

[10] Ahlberg G., Refsgaard L., Lundegaard P.R., Andreasen L., Ranthe M.F., Linscheid N., Nielsen J.B., Melbye M., Haunsø S., Sajadieh A. (2018). Rare truncating variants in the sarcomeric protein titin associate with familial and early-onset atrial fibrillation. Nat. Commun..

[11] Goodyer W.R., Dunn K., Caleshu C., Jackson M., Wylie J., Moscarello T., Platt J., Reuter C., Smith A., Trela A. (2019). Broad Genetic Testing in a Clinical Setting Uncovers a High Prevalence of Titin Loss-of-Function Variants in Very Early Onset Atrial Fibrillation. Circ. Genom. Precis. Med..

[12] Crotti L., Odening K.E., Sanguinetti M.C. (2020). Heritable arrhythmias associated with abnormal function of cardiac potassium channels. Cardiovasc. Res..

[13] Rosenbaum A.N., Agre K.E., Pereira N.L. (2020). Genetics of dilated cardiomyopathy: Practical implications for heart failure management. Nat. Rev. Cardiol..

[14] Richards S., Aziz N., Bale S., Bick D., Das S., Gastier-Foster J., Grody W.W., Hegde M., Lyon E., Spector E. (2015). Standards and guidelines for the interpretation of sequence variants: A joint consensus recommendation of the American College of Medical Genetics and Genomics and the Association for Molecular Pathology. Genet. Med..

[15] UK Biobank-UK Biobank. https://www.ukbiobank.ac.uk/.

[16] Bycroft C., Freeman C., Petkova D., Band G., Elliott L.T., Sharp K., Motyer A., Vukcevic D., Delaneau O., O’Connell J. (2018). The UK Biobank resource with deep phenotyping and genomic data. Nature.

[17] Li H., Durbin R. (2009). Fast and accurate short read alignment with Burrows-Wheeler transform. Bioinformatics.

[18] Tarasov A., Vilella A.J., Cuppen E., Nijman I.J., Prins P. (2015). Sambamba: Fast processing of NGS alignment formats. Bioinformatics. Bioinformatics.

[19] Poplin R., Chang P.C., Alexander D., Schwartz S., Colthurst T., Ku A., Newburger D., Dijamco J., Nguyen N., Afshar P.T. (2018). A universal SNP and small-indel variant caller using deep neural networks. Nat. Biotechnol..

[20] Kim S., Scheffler K., Halpern A.L., Bekritsky M.A., Noh E., Källberg M., Chen X., Kim Y., Beyter D., Krusche P. (2018). Strelka2: Fast and accurate calling of germline and somatic variants. Nat. Methods.

[21] Plagnol V., Curtis J., Epstein M., Mok K.Y., Stebbings E., Grigoriadou S., Wood N.W., Hambleton S., Burns S.O., Thrasher A.J. (2012). A robust model for read count data in exome sequencing experiments and implications for copy number variant calling. Bioinformatics.

[22] Sadedin S.P., Ellis J.A., Masters S.L., Oshlack A. (2018). Ximmer: A system for improving accuracy and consistency of CNV calling from exome data. GigaScience.

[23] Titin Variation in Dilated Cardiomyopathy—Cardiovascular Genetics & Genomics, Imperial College London. https://www.cardiodb.org/titin/titin_transcripts.php.

[24] Parnell L.D., Lindenbaum P., Shameer K., Dall’Olio G.M., Swan D.C., Jensen L.J., Cockell S.J., Pedersen B.S., Mangan M.E., Miller C.A. (2011). BioStar: An online question & answer resource for the bioinformatics community. PLoS Comput. Biol..

[25] Robinson J.T., Thorvaldsdóttir H., Winckler W., Guttman M., Lander E.S., Getz G., Mesirov J.P. (2011). Integrative Genomics Viewer. Nat. Biotechnol..

[26] Captur G., Arbustini E., Bonne G., Syrris P., Mills K., Wahbi K., Mohiddin S.A., McKenna W.J., Pettit S., Ho C.Y. (2018). Lamin and the heart. Heart.

[27] Gigli M., Begay R.L., Morea G., Graw S.L., Sinagra G., Taylor M.R., Granzier H., Mestroni L. (2016). A Review of the Giant Protein Titin in Clinical Molecular Diagnostics of Cardiomyopathies. Front. Cardiovasc. Med..

[28] Koelemen J., Gotthardt M., Steinmetz L.M., Meder B. (2021). RBM20-Related Cardiomyopathy: Current Understanding and Future Options. J. Clin. Med..

[29] Lin H., Koren S.A., Cvetojevic G., Girardi P., Johnson G.V.W. (2022). The role of BAG3 in health and disease: A “Magic BAG of Tricks”. J. Cell. Biochem..

[30] Chung I.M., Rajakumar G. (2016). Genetics of Congenital Heart Defects: The NKX2-5 Gene, a Key Player. Genes.

[31] Yoneda Z.T., Anderson K.C., Quintana J.A., O’Neill M.J., Sims R.A., Glazer A.M., Shaffer C.M., Crawford D.M., Stricker T., Ye F. (2021). Early-Onset Atrial Fibrillation and the Prevalence of Rare Variants in Cardiomyopathy and Arrhythmia Genes. JAMA Cardiol..

[32] Jordan E., Peterson L., Ai T., Asatryan B., Bronicki L., Brown E., Celeghin R., Edwards M., Fan J., Ingles J. (2021). Evidence-Based Assessment of Genes in Dilated Cardiomyopathy. Circulation.

[33] van Waning J.I., Caliskan K., Hoedemaekers Y.M., van Spaendonck-Zwarts K.Y., Baas A.F., Boekholdt S.M., van Melle J.P., Teske A.J., Asselbergs F.W., Backx A.P. (2018). Genetics, Clinical Features, and Long-Term Outcome of Noncompaction Cardiomyopathy. J. Am. Coll. Cardiol..

[34] Peled Y., Gramlich M., Yoskovitz G., Feinberg M.S., Afek A., Polak-Charcon S., Pras E., Sela B.A., Konen E., Weissbrod O. (2014). Titin mutation in familial restrictive cardiomyopathy. Int. J. Cardiol..

[35] Chalazan B., Mol D., Darbar F.A., Ornelas-Loredo A., Al-Azzam B., Chen Y., Chen Y., Tofovic D., Sridhar A., Alzahrani Z. (2021). Association of Rare Genetic Variants and Early-Onset Atrial Fibrillation in Ethnic Minority Individuals. JAMA Cardiol..

[36] Lazarte J., Laksman Z.W., Wang J., Robinson J.F., Dron J.S., Leach E., Liew J., McIntyre A.D., Skanes A.C., Gula L.J. (2021). Enrichment of loss-of-function and copy number variants in ventricular cardiomyopathy genes in ‘lone’ atrial fibrillation. EP Eur..

[37] Nelis M., Esko T., Mägi R., Zimprich F., Zimprich A., Toncheva D., Karachanak S., Piskáčková T., Balaščák I., Peltonen L. (2009). Genetic structure of Europeans: A view from the North-East. PLoS ONE.

[38] Hoorntje E.T., van Spaendonck-Zwarts K.Y., te Rijdt W.P., Boven L., Vink A., van der Smagt J.J., Asselbergs F.W., van Wijngaarden J., Hennekam E.A., Pinto Y.M. (2018). The first titin (c.59926 + 1G > A) founder mutation associated with dilated cardiomyopathy. Eur. J. Heart Fail..

[39] Andreasen L., Bertelsen L., Ghouse J., Lundegaard P.R., Ahlberg G., Refsgaard L., Rasmussen T.B., Eiskjær H., Haunsø S., Vejlstrup N. (2020). Early-onset atrial fibrillation patients show reduced left ventricular ejection fraction and increased atrial fibrosis. Sci Rep..

[40] Haggerty C.M., Damrauer S.M., Levin M.G., Birtwell D., Carey D.J., Golden A.M., Hartzel D.N., Hu Y., Judy R., Kelly M.A. (2019). Genomics-First Evaluation of Heart Disease Associated with Titin-Truncating Variants. Circulation.

[41] Yoneda Z.T., Anderson K.C., Ye F., Quintana J.A., O’Neill M.J., Sims R.A., Sun L., Glazer A.M., Davogustto G., El-Harasis M. (2022). Mortality Among Patients with Early-Onset Atrial Fibrillation and Rare Variants in Cardiomyopathy and Arrhythmia Genes. JAMA Cardiol..

[42] Wilde A.A.M., Semsarian C., Márquez M.F., Sepehri Shamloo A., Ackerman M.J., Ashley E.A., Sternick E.B., Barajas-Martinez H., Behr E.R., Bezzina C.R. (2022). European Heart Rhythm Association (EHRA)/Heart Rhythm Society (HRS)/Asia Pacific Heart Rhythm Society (APHRS)/Latin American Heart Rhythm Society (LAHRS) Expert Consensus Statement on the state of genetic testing for cardiac diseases. EP Eur..

