# Supplementary material for: Genetic Basis of Early Onset Atrial Fibrillation in Patients without Risk Factors
Source: J Cardiovasc Dev Dis. 2023 Feb 28;10(3):104. doi: 10.3390/jcdd10030104 (PMC10057774; doi:10.3390/jcdd10030104)
Supplement: Supplementary file 1 [file jcdd-10-00104-s001.zip › jcdd-2237663-supplementary.pdf]

Supplementary Table S1. Definition of early onset risk factor-free cases in UK Biobank database

**Inclusion codes ICD-10**	I48, I48.0, I48.1, I48.2, I48.9
**Exclusion ICD-10 codes**	I20, I20.0, I20.1, I20.8, I20.9, I21, I21.0, I21.1, I21.2, I21.3, I21.4, I21.9, I21.X, I22, I22.0, I22.1, I22.8, I22.9 I23, I23.0, I23.1, I23.2, I23.3, I23.4, I23.5, I23.6, I23.8, I24, I24.0, I24.1, I24.8, I24.9, I25, I25.0, I25.1, I25.2, I25.3, I25.4, I25.5, I25.6, I25.8, I25.9, Z95.1, Z95.5, Z95.8, Z95.9, I10, I11, I11.0, I11.9, I12, I12.0, I12.9, I13, I13.0, I13.1, I13.2, I13.9, I15, I15.0, I15.1, I15.2, I15.8, I15.9, I01.0, I01.1, I01.2, I01.8, I01.9, I02.0, I029, I05, I05.0, I05.1, I05.2, I05.8, I05.9, I06.0, I06.1, I06.2 I06.8, I06.9, I07, I07.0, I07.1, I07.2, I07.8, I07.9, I08, I08.0, I08.1, I08.2, I08.3, I08.8, I08.9, I09, I09.0, I09.1, I09.2, I09.8, I09.9, I33, I33.0, I33.9, I34, I34.0, I34.1, I34.2, I34.8, I34.9, I35, I35.0, I35.1, I35.2, I35.8, I35.9, I36, I36.0, I36.1, I36.2, I36.8, I36.9, I37, I37.0, I37.1, I37.2, I37.8, I37.9, I38, I39, I39.0, I39.1, I39.2, I39.3, I39.4, I39.8, Z95, Z95.2, Z95.3, Z95.4, I43, I43.0, I43.1, I43.2, I43.8, I42, I42.0, I42.1, I42.2, I42.3, I42.4, I42.5, I42.6, I42.7, I42.8, I42.9, I40, I40.0, I40.1, I40.8, I40.9, I41, I41.0, I41.1, I41.2, I41.8, I50, I50.0, I50.1, I50.9, I51.0, I51.1, I51.2, Q20, Q20.0, Q20.1, Q20.2, Q20.3, Q20.4, Q20.5, Q20.6, Q20.8, Q20.9, Q21, Q21.0, Q21.1, Q21.2, Q21.3, Q21.4, Q21.8, Q21.9, Q22, Q22.0, Q22.1, Q22.2, Q22.3, Q22.4, Q22.5, Q22.6, Q22.8, Q22.9, Q23, Q23.0, Q23.1, Q23.2, Q23.3, Q23.4, Q23.8, Q23.9, Q24, Q24.0, Q24.1, Q24.2, Q24.3, Q24.4, Q24.5, Q24.6, Q24.8, Q24.9, Q25, Q25.0, Q25.1, Q25.2, Q25.3, Q25.4, Q25.5, Q25.6, Q25.7, Q25.8, E10, E10.0, E10.1, E10.2, E10.3, E10.4, E10.5, E10.6, E10.7, E10.8, E10.9, E11, E11.0, E11.1, E11.2, E11.3, E11.4, E11.5, E11.6, E11.7, E11.8, E11.9, E12, E12.0, E12.1, E12.2, E12.3, E12.4, E12.5, E12.6, E12.7, E12.8, E12.9, E13, E13.0, E13.1, E13.2, E13.3, E13.4, E13.5, E13.6, E13.7, E13.8, E13.9, E14, E14.0, E14.1, E14.2, E14.3, E14.4, E14.5, E14.6, E14.7, E14.8, E14.9, E00.9, E01, E01.0, E01.1, E01.2, E01.8, E02, E03, E03.0, E03.1, E03.2, E03.3, E03.4, E03.5, E03.8, E03.9, E04, E04.0, E04.1, E04.2, E04.8, E04.9, E05, E05.0, E05.1, E05.2, E05.3, E05.4, E05.5, E05.8, E05.9, E06, E06.0, E06.1, E06.2, E06.3, E06.4, E06.5, E06.9, E07, E07.0, E07.1, E07.8, E07.9, J41, J410, J41.0, J41.1, J41.8, J42, J43, J43.0, J431, J43.1, J43.2, J43.8, J43.8, J43.9, J44, J44.0, J44.1, J44.8, J44.9, J45, J45.0, J45.1, J45.8, J45.9, J46, G47.3, N18, N18.0, N18.1, N18.2, N18.3, N18.4, N18.5, N18.8, N18.9, N19, M30

	M30.0, M30.1, M30.2, M30.3, M30.8, M31, M31.0, M31.1, M31.2, M31.3, M31.4, M31.5, M31.6, M31.7, M31.8, M31.9, M32, M32.0, M32.1, M32.8, M32.9, M32.90, M33, M33.0, M33.1, M33.2, M33.9, M34, M34.0, M34.1, M34.2, M34.8, M34.9, M35, M35.0, M35.1, M35.2, M35.3, M35.4, M35.5, M35.6, M35.8, M35.9, M35.99, M36, M36.0, M36.1, M36.2, M36.3, M36.4, M36.8, M45, M45.X0, M45.X1, M45.X2, M45.X3, M45.X4, M45.X5, M45.X6, M45.X7, M45.X8, M45.X9, F10, F10.0, F10.1, F10.2, F10.3, F10.4, F10.5, F10.6, F10.7, F10.8, F10.9, G31.2, G62.1, I42.6, K29.2, K70, K70.1, K70.2, K70.3, K70.4, K70.9, K86.0, T51.0
**Exclusion OPCS codes**	K40, K40.1, K40.2, K40.3, K40.4, K40.8, K40.9, K41, K41.1, K41.2, K41.3, K41.4, K41.8, K41.9, K43, K43.1, K43.2, K43.3, K43.4, K43.8, K43.9, K44, K44.1, K44.2, K44.8, K44.9, K45, K45.1, K45.2, K45.3, K45.4, K45.5, K45.6, K45.8, K45.9, K46, K46.1, K46.2, K46.3, K46.4, K46.5, K46.8, K46.9, K75.1, K75.2, K75.3, K75.4, K75.8, K75.9, K25.1, K25.2, K25.3, K25.4, K25.5, K25.8, K25.9, K26, K26.1, K26.2, K26.3, K26.4, K26.5, K26.8, K26.9, K27, K27.1, K27.2, K27.3, K27.4, K27.5, K27.6, K27.8, K27.9, K28, K28.1, K28.2, K28.3, K28.4, K28.5, K28.8, K28.9, K29, K29.1, K29.2, K29.3, K29.4, K29.5, K29.6, K29.7, K29.8, K29.9, K30, K30.1, K30.2, K30.3, K30.4, K30.5, K30.8, K30.9, K31, K31.1, K31.2, K31.3, K31.4, K31.5, K31.8, K31.9, K32, K32.1, K32.2, K32.3, K32.4, K32.8, K32.9, K34, K34.1, K34.2, K34.3, K34.4, K34.5, K34.6, K34.8, K34.9, K36, K36.1, K36.2, K36.8, K36.9, K04.1, K04.2, K04.3, K04.4, K04.5, K04.6, K04.8, K04.9, K05, K05.1, K05.2, K05.8, K05.9, K06, K06.1, K06.2, K06.3, K06.4, K06.8, K06.9, K07, K07.1, K07.2, K07.3, K07.8, K07.9, K08, K08.1, K08.2, K08.3, K08.4, K08.8, K08.9, K09, K09.1, K09.2, K09.3, K09.4, K09.5, K09.6, K09.8, K09.9, K10, K10.1, K10.2, K10.3 K10.4, K10.5, K10.8, K10.9, K11, K11.1, K11.2, K11.3, K11.4, K11.5, K11.6, K11.7, K11.8, K11.9, K12.1, K12.2, K12.3, K12.4, K12.5, K12.8, K12.9, K14.1, K14.2, K14.3, K14.4, K14.5, K14.8, K14.9, K17.1, K17.2, K17.3, K17.4, K17.5, K17.6, K17.7, K17.8, K17.9, K18.1, K18.2, K18.3, K18.4, K18.5, K18.6, K18.7, K18.8, K18.9, K19, K19.1, K19.2, K19.3, K19.4, K19.5, K19.6, K19.8, K19.9, K20, K20.1, K20.2, K20.3, K20.4, K20.8, K20.9, K22.1, K22.2, K22.8, K22.9, K23, K23.1, K23.2, K23.3, K23.5, K23.6, K23.8, K23.9, K24, K24.1, K24.2, K24.3, K24.4, K24.5, K24.6, K24.7, K24.8, K24.9, M01, M01.1, M01.2, M01.3, M01.4, M01.5, M01.8, M01.9

**Age at AF onset**	<65 years
**Age at recruitment**	< 65 years
**Body mass index**	<25 kg/m^2

ICD-10 – international classification of disease
OPCS – Office of Population Censuses and Surveys Classification of Surgical Operations and Procedures

Supplementary Table S2. List of genes included into analysis

AARS2, ABCC6, ABCC9, ACAD9, ACADVL, ACTA1, ACTC1, ACTN2, ACVR2B, AGK, AGL, AGPAT2, AKAP9, ALMS1, ANK2, ANKRD1, ANO5, APOA1, ARSB, ATPAF2, B3GAT3, BAG3, BCS1L, BRAF, BSCL2, CACNA1C, CACNA1D, CACNA2D1, CACNB2, CALM1, CALM3, CALR3, CAPN3, CASQ2, CAV3, CBL, CFC1, CHRM2, COA5, COQ2, COX10, COX15, COX6B1, CPS1, CPT2, CRELD1, CRYAB, CSRP3, CTNNA3, DBH, DES, DHCR7, DLD, DMD, DNAJC19, DOLK, DPM3, DPP6, DSC2, DSG2, DSP, DTNA, DYSF, ELAC2, ELN, EMD, ETFA, ETFB, ETFDH, EYA4, FAH, FASTKD2, FHL1, FHL2, FKRP, FKTN, FLNA, FLNC, FOXD4, FOXRED1, FXN, GAA, GALNS, GATA4, GATA6, GATAD1, GBE1, GDF1, GFM1, GJA1, GJA5, GLA, GLB1, GLRA1, GNPTAB, GNS, GPD1L, GSK3B, GUSB, GYG1, HADHA, HADHB, HAMP, HAND1, HCN4, HFE, HGSNAT, HRAS, IDH2, IDS, IDUA, ILK, IRX3, JAG1, JPH2, JUP, KCNA5, KCND3, KCNE1, KCNE2, KCNE3, KCNE5, KCNH2, KCNJ2, KCNJ5, KCNJ8, KCNN3, KCNQ1, KRAS, LAMA2, LAMA4, LAMP2, LDB3, LEFTY2, LIAS, LMNA, LRPPRC, MAP2K1, MAP2K2, MLYCD, MMACHC, MRPL3, MRPS22, MTO1, MYBPC3, MYH6, MYH7, MYL2, MYL3, MYLK2, MYOT, MYOZ2, MYPN, NAGLU, NDUFA1, NDUFA10, NDUFA11, NDUFA2, NDUFA4, NDUFA6, NDUFA9, NDUFAF1, NDUFAF2, NDUFAF3, NDUFAF4, NDUFAF5, NDUFAF6, NDUFB3, NDUFS1, NDUFS2, NDUFS3, NDUFS4, NDUFS6, NDUFS7, NDUFS8, NDUFV1, NDUFV2, NEXN, NF1, NKX2-5, NODAL, NOS1AP, NOTCH1, NOTCH2, NPPA,

NR2F2, NRAS, NUBPL, NUP155, OBSCN, PCCA, PCCB, PDHA1, PDLIM3, PHKA1, PHOX2B, PITX2, PKP2, PLEC, PLN, PMM2, PNPLA2, PRKAG2, PSEN1, PSEN2, PTPN11, RAB3GAP2, RAF1, RBM20, RYR2, SALL4, SCN10A, SCN1B, SCN2B, SCN3B, SCN4B, SCN5A, SCNN1B, SCNN1G, SCO1, SCO2, SDHA, SDHAF1, SDHD, SELENON, SGCA, SGCB, SGCD, SGCG, SGSH, SHOC2, SHROOM3, SLC22A5, SLC25A20, SLC25A3, SLC25A4, SLC40A1, SLC4A3, SNTA1, SOS1, SPRED1, SURF1, SYNE2, TAB2, TACO1, TAZ, TBX20, TBX5, TCAP, TFR2, TGFB3, TMEM43, TMEM70, TMPO, TNNC1, TNNI3, TNNI3K, TNNT2, TPM1, TRDN, TRIM32, TRPM4, TSFM, TTC19, TTN, TTR, UQCRB, VCL, VCP, VPS13A, XK, ZFHX3, ZIC3
